# HO-1 Induction by CO-RM2 Attenuates TNF-****α****-Induced Cytosolic Phospholipase A_2_ Expression via Inhibition of PKC****α****-Dependent NADPH Oxidase/ROS and NF-****κ****B

**DOI:** 10.1155/2014/279171

**Published:** 2014-01-29

**Authors:** Pei-Ling Chi, Chun-Ju Liu, I-Ta Lee, Yu-Wen Chen, Li-Der Hsiao, Chuen-Mao Yang

**Affiliations:** ^1^Department of Physiology and Pharmacology and Health Aging Research Center, College of Medicine, Chang Gung University, Kwei-San, Tao-Yuan 333, Taiwan; ^2^Department of Anesthetics, Chang Gung Memorial Hospital at Linkou and College of Medicine, Chang Gung University, Kwei-San, Tao-Yuan 333, Taiwan

## Abstract

Rheumatoid arthritis (RA) is characterized by chronic inflammatory infiltration of the synovium and elevation of proinflammatory cytokines. Cytosolic phospholipase A_2_ (cPLA_2_) is involved in the development of inflammatory diseases. Heme oxygenase-1 (HO-1) has been shown to possess anti-inflammatory properties. The objective of the study was to investigate the detailed mechanisms of TNF-**α**-induced cPLA_2_ expression and to determine whether carbon monoxide releasing molecule-2 (CO-RM2) suppresses TNF-**α**-induced expression of NF-**κ**B-related proinflammatory genes, including cPLA_2_, via HO-1 induction in RA synovial fibroblasts (RASFs). Here, we reported that TNF-**α**-induced cPLA_2_ expression was mediated through TNFR1/PKC**α**-dependent signaling pathways, including NADPH oxidase (NOX) activation/ROS production and NF-**κ**B activation. CO-RM2 significantly suppressed TNF-**α**-induced cPLA_2_ expression by inhibiting the ROS generation and the phosphorylation of NF-**κ**B p65 and IKK**α**/**β**, but not the phosphorylation of p38 MAPK and JNK1/2. These results were further confirmed by a ChIP assay to detect the NF-**κ**B DNA-binding activity. Our results demonstrated that induction of HO-1 by CO-RM2 exerted anti-inflammatory and antioxidant effects which were required in concert to prevent the activation of NF-**κ**B leading to induction of various inflammatory genes implicated in the pathogenesis of RA.

## 1. Introduction

Synovial cells appear to be involved in both the inflammatory cell infiltration of the synovium and progressive synovial inflammation, resulting in irreversible joint destruction [1]. Once activated, synovial cells produce TNF-*α* and IL-1*β* that are involved in sustaining regulatory feedback loops and induce the expression of inflammatory mediators [2]. Cytosolic phospholipase A_2_ (cPLA_2_) is responsible for liberation of arachidonic acid from the sn-2 position of membrane phospholipids, leading to prostaglandin and leukotriene biosynthesis [3]. It has been shown that cPLA_2_ is upregulated by IL-1*β* in human rheumatoid arthritis synovial fibroblasts (RASFs) [4]. cPLA_2_-deficient mice show markedly reduced synovial inflammation and severity of disease in collagen-induced arthritis [5]. Therefore, suppression of cPLA_2_ expression and PGE_2_ production are considered important targets of therapy for rheumatoid arthritis (RA).

The RA synovium is exposed to ROS produced by synovial fibroblasts, which are implicated in the pathogenesis of RA [6]. One of the principal sources of superoxide is NADPH oxidase (NOX); this NOX complex is composed of two membrane-located subunits p22^phox^ and NOX2 and a complex made up of p40^phox^,  p47^phox^, and p67^phox^,   localized in the cytoplasm [7, 8]. Evidence has indicated that activation of NOX involves phosphorylation of p47^phox^ that initiates assembly of the cytoplasmic components and translocation to the membrane for complete association with NOX and functioning of the oxidase [9–11], suggesting that p47^phox^ is a regulatory subunit of the membrane NOX. In synoviocytes, NOX2 could be particularly responsible for superoxide production by cytokines [12]. Accumulating evidence also indicates that ROS act as second messengers in the activation of NF-*κ*B [11], leading to induction of various inflammatory genes [13, 14]. However, the molecular mechanisms between NOX/ROS and NF-*κ*B activation by which TNF-*α* induced cPLA_2_ expression remain unknown.

Heme oxygenase 1 (HO-1) is induced by oxidative stress and different stimuli, which acts as a host defense mechanism due to its antioxidant and anti-inflammatory effects [11, 15, 16]. The precise mechanisms for HO-1-based protection are not yet completely understood. Accumulating evidence has demonstrated that the protective effects of HO-1 may be mediated through its by-products including carbon monoxide (CO), biliverdin/bilirubin, and free iron. Among the HO-1 by-products, CO has been shown to suppress the expression of LPS-induced proinflammatory cytokines and to increase LPS-induced expression of IL-10 in macrophages, suggesting that CO is involved in the anti-inflammatory action of HO-1 [17]. Recently, metal carbonyl compounds have been identified as CO-releasing molecules (CORMs) with the potential to facilitate the pharmaceutical use of CO by delivering it to tissues and organs [18]. These compounds have been shown to attenuate leukocytes sequestration in the liver and lung tissues by interfering with NF-*κ*B activation and ICAM-1 expression and therefore suppressing endothelial cells proadhesive phenotype [19]. Therefore, we hypothesized that HO-1 mediates its salutary effects in TNF-*α*-induced inflammatory joints via downregulation of cPLA_2_. To test this, we used tricarbonyldichlororuthenium (II) dimer ([Ru(CO)_3_Cl_2_]_2_, CORM-2) as a CO-releasing molecule and HO-1 inducer.

Here, we reported that TNFR1/PKC*α*-dependent activation of NF-*κ*B was mediated through phosphorylation of IKK*α*/*β* and NF-*κ*B p65 and NOX/ROS generation, which was required for induction of cPLA_2_ in TNF-*α*-challenged RASFs. On the other hand, CORM-2 increased HO-1 expression and significantly downregulated TNF-*α*-induced cPLA_2_ expression. The possible mechanisms by which CORM-2 exerts protective effects were mediated through suppression of NOX/ROS production and IKK*α*/*β* and p65 phosphorylation in response to TNF-*α*. Our findings provide a new insight into the mechanisms by which CORM-2 exerts antioxidant and anti-inflammatory effects in RA.

## 2. Materials and Methods

### 2.1. Materials

Diphenyleneiodonium chloride (DPI), Gö6976, U0126, SB202190, SP600125, and helenalin were obtained from Biomol (Plymouth Meeting, PA). Apocynin was from ChromaDex (Santa Ana, CA). N-acetylcysteine (NAC), tricarbonyldichlororuthenium (II) dimer (CORM-2) and ruthenium (III) chloride (inactive CORM-2) were purchased from Sigma (St. Louis, MO). Anti-GAPDH antibody was obtained from Biogenesis (Boumemouth, UK). Anti-p47^phox^, anti-HO-1, anti-Gs*α*, anti-gp91^phox^, anti-*β*-actin, and anti-cPLA_2_ antibodies were from Santa Cruz (Santa Cruz, CA). Anti-phospho-p38 MAPK, anti-phospho-JNK1/2, anti-phospho-PKC*α*/*β*II, anti-phospho-p65, and anti-phospho-IKK*α*/*β* antibodies were from Cell Signaling (Danvers, MA). Dihydroethidium (DHE) was from Molecular Probes (Eugene, OR).

### 2.2. Isolation and Culture of Human Synovial Fibroblasts

RASFs were obtained from 29 patients with RA who underwent knee or hip surgery. Informed consent was obtained from all patients, and the experimental protocol was approved by the Institutional Review Board, Chang Gung Memorial Hospital. RASFs were isolated, cultured, and characterized as previously described [20, 21]. Experiments were performed using cells from passages 3 to 6.

### 2.3. Animals

Male ICR mice aged 4–6 weeks were purchased from National Taiwan University, College of Medicine, Laboratory Animal Center. Mice were maintained under conditions consistent with the Guidelines of the Animal Care Committee of Chang Gung University as well as the Guide for the Care and Use of Laboratory Animals of the National Research Council in the USA. Mice were anesthetized by intraperitoneal injection of 200 *μ*L of pentobarbital sodium (5 mg/mL). Mice were given an intraarticular injection of CORM-2 (8 *μ*g/kg of body weight) or phosphate-buffered saline (PBS) 16 hours before treatment with TNF-*α* (30 *μ*g/kg of body weight) and were sacrificed after 24 hours.

### 2.4. Immunohistochemical Staining

Immunohistochemical staining was performed on the serial sections of the ankle joints, which were deparaffinized, rehydrated, and washed with PBS. Nonspecific binding was blocked by preincubation with PBS containing 5 mg/mL of BSA for 1 h at room temperature. The section was incubated with anti-cPLA_2_ or anti-HO-1 at 37°C for 1 h and then with an anti-rabbit horseradish peroxidase Ab at room temperature for 1 h. Bound Abs were detected by incubation in 0.5 mg/mL of 3,3′-diaminobenzidine/0.01% hydrogen peroxide in 100 mM Tris-HCl buffer, as chromogen (Vector Lab., Burlingame, CA). The second section was incubated with an anti-vimentin Ab for the positive localization and identification of synovial fibroblasts. Images were obtained under a light microscopy at a magnification ×200 or ×400. The quantitative data of immunohistochemical staining were calculated the percentage of cPLA_2_-positive cells over the vimentin-positive cells under the microscopic field, using ImageJ software.

### 2.5. Immunofluorescence Staining

Cells were plated on 6-well culture plates with coverslips, shifted to serum-free DMEM-Ham's F-12 for 24 hours, and then incubated with TNF-*α*. Cells were fixed, permeabilized, and stained using an anti-p65 antibody as previously described [21]. The images were collected on a fluorescence microscope (Axiovert 200 M, Zeiss) using a 100x objective.

### 2.6. Western Blot Analysis

Growth-arrested RASFs were incubated with TNF-*α* for the indicated time intervals. The cells were washed, scraped, collected, and centrifuged at 45000 ×g at 4°C for 1 h to yield the whole cell extract, as previously described [21]. Samples were denatured, subjected to SDS-PAGE using a 12% running gel, and transferred to nitrocellulose membrane. Membranes were incubated with an anti-cPLA_2_ antibody for 24 h and then incubated with an anti-mouse horseradish peroxidase antibody for 1 h. The immunoreactive bands were detected by ECL reagents.

### 2.7. Transient Transfection with siRNAs

Human siRNAs of PKC*α*, p47^phox^, p38, JNK1, and scrambled were from Sigma (St. Louis, MO). Transient transfection of siRNAs was performed using Metafectene transfection reagent from Biontex Lab (GmbH, Planegg/Martinsried, Germany) according to the manufacturer's instructions. The transfection mixture was diluted into 500 *μ*L of DMEM/F-12 medium with 10% FBS and antibiotics and added directly to the cells. After 16 h transfection, the medium was replaced with serum-free DMEM/F-12 for 24 h. Cell lysates prepared from RASFs challenged with TNF-*α* were analyzed by Western blot.

### 2.8. Real-Time Quantitative PCR Analysis

RNA was extracted using TRIzol and first-strand cDNA synthesis was done with 1 *μ*g of total RNA using Superscript II reverse transcriptase (Invitrogen) according to the manufacturer's protocols. The primers and probes used for real-time PCR of human cPLA_2_ and GAPDH were obtained from Applied Biosystems (Foster City, CA). Each PCR reaction (20 *μ*L) contained 100 ng of cDNA, PCR master mix, and premade TaqMan gene expression assay components containing a FAM reporter dye at the 5′-end of the TaqMan probe and a nonfluorescent quencher (NFQ) at the 3′-end of the probe. Human GAPDH was used as a control to verify the quality of cDNA template. Real-time PCR was performed and analyzed by an ABI StepOnePlus QPCR instrument (Foster City, CA).

### 2.9. Measurement of Intracellular ROS Accumulation

At the indicated time after stimulation, dihydroethidium (DHE, 5 *μ*M) was added to the medium and incubated for 30 minutes at 37°C. The cells were washed with PBS and DHE fluorescent images of RASFs were visualized on a fluorescence microscope using a 20x objective (Zeiss, Axiovert 200 M). The average fluorescence intensity values for 20–30 cells in 3 different examinations were calculated using ImageJ software.

### 2.10. NADPH Oxidase Activity

NADPH oxidase activity was measured by lucigenin-enhanced chemiluminescence in a 50 mM phosphate buffer (buffer A), containing 1 mM EGTA, protease inhibitors, 150 mM sucrose, 10 *μ*M lucigenin (Sigma), and 10 *μ*M NADPH as substrate [22]. Quiescent cells were starved by serum deprivation for 24 hours and treated as indicated, washed twice with ice-cold phosphate-buffered saline (PBS), and harvested. After low spin centrifugation, the pellet was resuspended in ice-cold buffer A, lacking lucigenin and substrate. The total protein concentration was determined using a BCA protein assay kit (Pierce, USA) and adjusted to 1 mg/mL. 100 *μ*L aliquots of the protein sample were measured over 10 min in quadruplicate using NADPH as substrate in an Appliskan luminometer (Thermo) in out-of-coincidence mode.

### 2.11. Preparation and Analysis of Cell Fractions

Cells were harvested and then washed twice with ice-cold PBS, 300 *μ*L of homogenization buffer A (20 mM Tris-HCl (pH 8.0), 10 mM EGTA, 2 mM EDTA, 2 mM DTT, 1 mM PMSF, 25 *μ*g/mL aprotinin, and 10 *μ*g/mL leupeptin) was added to each dish, and the cells were scraped into a 1.5 mL tube with a rubber policeman. The suspension was sonicated for 10 s at output 4 with a sonicator (Ultrasonics) and centrifuged at 5000 ×g for 15 min at 4°C to pellet nuclei and other fragments. The supernatant can be retained as the cytoplasmic fraction and was further centrifuged at 15000 ×g for 60 min at 4°C to yield the pellet (membrane fraction) and the supernatant (cytosolic fraction). Gs*α* was used as marker protein for membrane fraction.

### 2.12. Coimmunoprecipitation Assay

Cell lysates containing 1 mg of protein were incubated with 2 *μ*g of anti-PKC*α* or anti-TNFR1 antibody at 4°C for 24 h, and then 10 *μ*L of 50% protein A-agarose beads was added and mixed at 4°C for 24 h. The immunoprecipitates were collected and washed three times with lysis buffer without Triton X-100. 5X Laemmli buffer was added, subjected to electrophoresis on 12% SDS-PAGE, and then blotted using an anti-PKC*α* or anti-TNFR1 antibody.

### 2.13. Measurement of cPLA_2_ and NF-*κ*B Promoter Activities

For construction of the cPLA_2_-promoter-Luc plasmid, a human cPLA_2_ promoter region (~1674 bp) was PCR amplified from human genomic DNA and inserted between luciferase gene and SV40 late poly(A) signal coding regions of luciferase plasmid pGL3 as the wild type cPLA_2_ promoter plasmids. The cPLA_2_ promoter region was amplified by conventional PCR using the following primers: the forward primer 5′-GGGGTACCAGAACGAACATGCCCTGCAGTATAGA-3′ and the reverse primer 5′-GGAAGCTTGCTGACTTTAAGCAGCGAGG-3′. The DNA fragments were directly subcloned into pGL3 using KpnI and HindIII. The vector sequence was confirmed by DNA sequencing and amplified by using QIAGEN plasmid DNA preparation kits. The pNF-*κ*B-Luc (Clontech) or cPLA_2_ promoter activity was determined as previously described [21]. Firefly promoter luciferase activities were standardized for *β*-galactosidase activity.

### 2.14. Chromatin Immunoprecipitation (ChIP) Assay

ChIP assay was performed as previously described [21]. Soluble chromatin was immunoprecipitated using an anti-p65 antibody. The purified DNA was subjected to PCR amplification using primers specific for the region containing the NF-*κ*B binding site present in the cPLA_2_ promoter: 5′-GAGACGGAGTCTCGCTCTGT-3′  (sense) and 5′-GTGGCTCACGCCTGTAATCC-3′(antisense). PCR fragments were analyzed on 2% agarose in 1X Tris-acetate-EDTA gel containing ethidium bromide.

### 2.15. Measurement of PGE_2_ Release

Cells were treated with TNF-*α* (30 ng/mL) for 16 hours in the presence or absence of the indicated inhibitors. The media were collected, and PGE_2_ was assayed using a PGE_2_ enzyme immunoassay kit (Cayman Chemical).

### 2.16. Statistical Analysis of Data

All data are representative of at least three independent experiments and comparisons of ≥3 populations were made using GraphPad Prism Program (GraphPad Software, Inc.). Data were expressed as the mean ± SEM and analyzed by one-way ANOVA followed with Tukey's post hoc test. Significant differences between the compared groups are indicated: *P < 0.05; ^#^ *P* < 0.01.

## 3. Results

### 3.1. Overexpression of HO-1 by CORM-2 Attenuates TNF-*α*-Induced cPLA_2_ Expression

**Figure 1:**
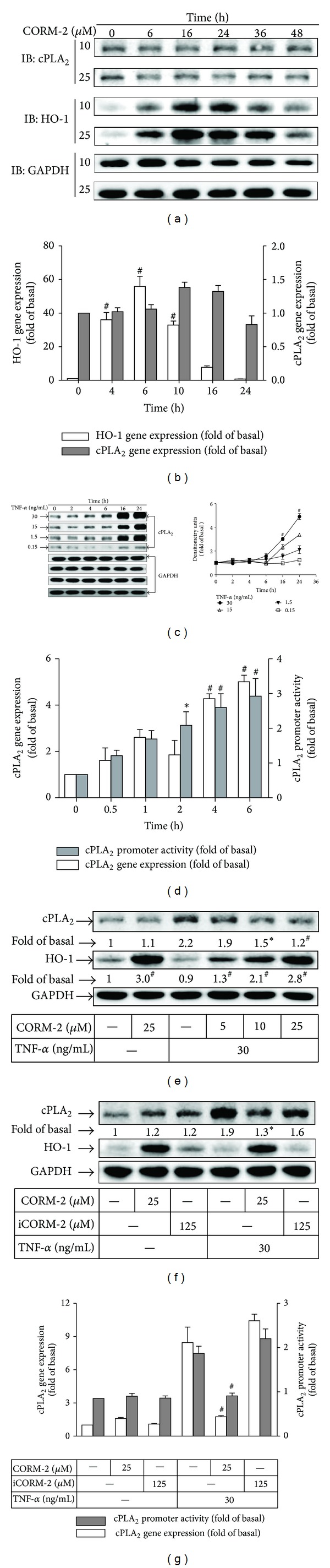
CORM-2 enhances HO-1 expression and modulates TNF-*α*-induced cPLA_2_ expression in RASFs. RASFs were treated with (a) different concentrations or (b) 25 *μ*M of CORM-2 for the indicated time intervals. (c) RASFs were incubated with various concentrations of TNF-*α* for the indicated time intervals. (d) Cells were incubated with TNF-*α* (30 ng/mL) for the indicated time intervals. The mRNA levels of cPLA_2_ and cPLA_2_-Luc promoter were measured. (e) Cells were pretreated with different concentrations of CORM-2 for 16 h and then incubated with TNF-*α* for 16 h. Cells were pretreated with (f) CORM-2 or iCORM-2 or (h) were transfected with scrambled (scrb) or HO-1 siRNA and then incubated without or with TNF-*α* for 16 h. ((a), (c), (e), (f), and (h)) The cell lysates were analyzed by Western blotting using an anti-cPLA_2_, anti-HO-1, or anti-GAPDH (control). ((g), open bars) RASFs were pretreated without or with CORM-2, or iCORM-2 for 16 h, and incubated with TNF-*α* (30 ng/mL) for 6 h. cPLA_2_ mRNA was analyzed by quantitative real-time PCR. ((g), shaded bars) RASFs were transfected with a cPLA_2_-Luc reporter gene, pretreated without or with CORM-2 or iCORM-2 for 16 h, and incubated with TNF-*α* (30 ng/mL) for 6 h. Promoter luciferase activity was analyzed. All analyses were performed on samples from 4 RA patients. Results are representative of 3 independent experiments. Values are the mean ± SEM. ((b)–(d)) *P < 0.05; ^#^ *P* < 0.01, as compared with the cells exposed to vehicle alone; ((e)–(g)) *P < 0.05; ^#^ *P* < 0.01 as compared with the cells exposed to TNF-*α* alone.

First, we examined the effect of CORM-2 on HO-1 expression in human RASFs. Treatment of human RASFs with CORM-2 resulted in an increase in HO-1 protein and mRNA expression, but not cPLA_2_ (Figures 1(a) and 1(b)). Next, we found that TNF-*α* induced cPLA_2_ protein and mRNA expression, and promoter activity in a time- and concentration-dependent manner (Figures 1(c) and 1(d)). In our previous study, overexpression of HO-1 in human tracheal smooth muscle cells has been shown to inhibit expression of VCAM-1 and ICAM-1 induced by cytokines [11]. Hence, we examined the role of HO-1 in TNF-*α*-induced cPLA_2_ expression. To determine whether HO-1 protein overexpression can downregulate cPLA_2_ expression, we pretreated human RASFs with CORM-2 for 16 h and then incubated with TNF-*α* for 16 h. cPLA_2_ expression was significantly induced by TNF-*α*, which was attenuated by HO-1 induction by CORM-2 in a concentration-dependent manner (Figure 1(e)). Further, we characterized the expression of HO-1 specifically induced by CORM-2. As shown in Figure 1(f), incubation with inactive form of CORM-2 (iCORM-2) failed to induce HO-1 expression and to reduce TNF-*α*-induced cPLA_2_ expression. Consistently, TNF-*α*-stimulated cPLA_2_ mRNA expression and promoter activity were also reduced by the pretreatment of CORM-2 (Figure 1(g)). These results indicated that CORM-2-induced HO-1 expression exerted inhibitory effects on TNF-*α*-induced cPLA_2_ expression in RASFs.

### 3.2. TNF-*α*-Mediated Recruitment of PKC*α* to TNFR1 Complex Is Involved in cPLA_2_ Induction

**Figure 2:**
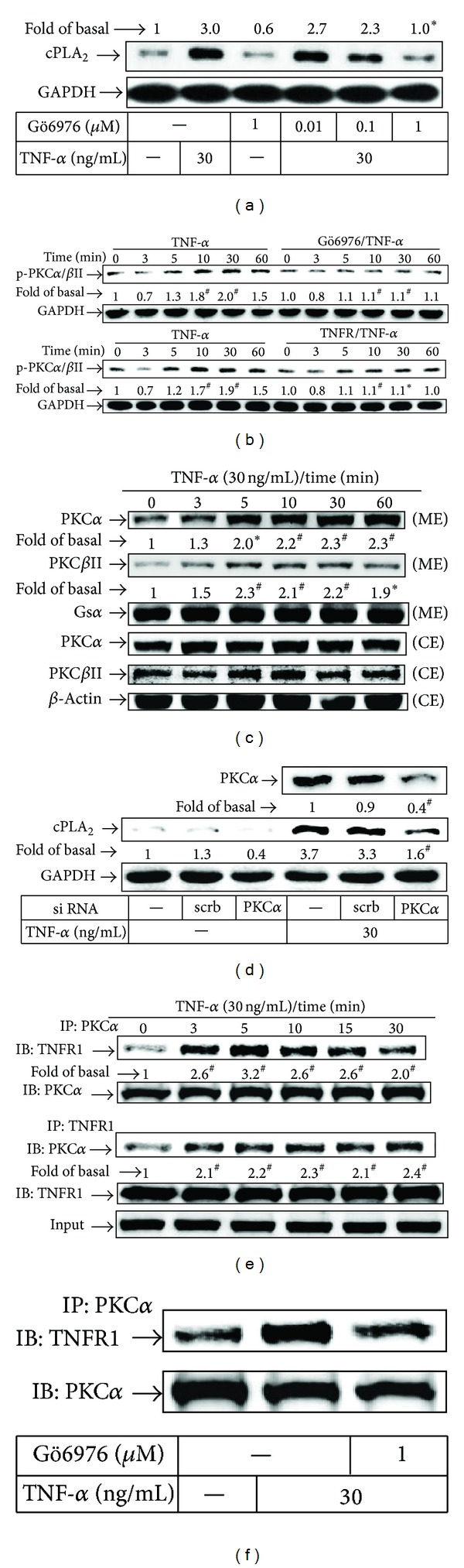
TNF-*α*-induced cPLA_2_ expression is mediated via the formation of a TNFR1/PKC*α* complex. RASFs were pretreated with (a) Gö6976 for 1 h or (d) transfected with PKC*α* or scrambled (scrb) siRNA and then incubated with TNF-*α* for 16 h. (b) Cells were pretreated without or with Gö6976 (1 *μ*M) or an anti-TNFR1 neutralizing Ab (1 *μ*g/mL) for 1 h and then incubated with TNF-*α* (30 ng/mL) for the indicated time intervals. (c) RASFs were stimulated with TNF-*α* for the indicated time intervals. The membrane (ME) and cytosolic extracts (CE) were prepared. The cell fractions were analyzed by Western blot using the indicated antibodies and anti-Gs*α* and *β*-actin used as internal controls for ME and CE, respectively. ((e) and (f)) RASFs were pretreated with or without (f) Gö6976 (1 *μ*M) for 1 h and stimulated with TNF-*α* for the indicated time intervals (e) or for 5 min (f). RASFs were immunoprecipitated (IP) using an anti-PKC*α* (top) or anti-TNFR1 (bottom) antibody. The cell lysates were immunoprecipitated and analyzed by Western blotting using an anti-PKC*α* or anti-TNFR1 antibody. Input represents whole cell extracts before immunoprecipitation. All analyses were performed on samples from 4 RA patients. Results are representative of 3 independent experiments. Values are the mean ± SEM. In (b) (left, control part), (c), and (e), *P < 0.05; ^#^ *P* < 0.01 versus vehicle alone. In (a), (b) (right, experimental part), and (d), *P < 0.05; ^#^ *P* < 0.01 versus TNF-*α* alone.

Most of TNF-*α* effects are elicited through TNFR1. In addition, PKC*α* is a critical regulator in TNF-*α*/TNFR1-mediated signaling [23]. In this study, we explored whether PKC*α* could regulate TNF-*α*-induced cPLA_2_ expression. As illustrated in Figure 2(a), TNF-*α*-induced cPLA_2_ expression was inhibited by pretreatment with an inhibitor of PKC*α*/*β*, Gö6976. Moreover, TNF-*α* time-dependently stimulated PKC*α*/*β*II phosphorylation at Thr^638/641^ with a maximal response within 10 to 60 min (Figure 2(b)). Pretreatment with Gö6976 attenuated TNF-*α*-stimulated PKC*α*/*β*II phosphorylation during the period of observation (Figure 2(b), upper panel). We also found that TNF-*α* induced PKC*α*/*β*II phosphorylation via TNFR1 by using a TNFR1 neutralizing antibody (TNFR1 nAb) (Figure 2(b), lower panel). We further demonstrated that TNF-*α* stimulated PKC*α* and PKC*β*II translocation from the cytosol to the membrane (Figure 2(c)). To further ensure the role of PKC*α* in TNF-*α*-induced cPLA_2_ expression, as shown in Figure 2(d), transfection with PKC*α* siRNA downregulated the expression of total PKC*α* protein and then markedly inhibited cPLA_2_ expression induced by TNF-*α*. The interplay between TNFR1 and PKC isoforms signaling pathways in inflammatory response is reflected in the expression and regulation of key proteins [24]. We next examined the interaction between TNFR1 and PKC*α* in TNF-*α*-stimulated RASFs. We found that TNF-*α* time-dependently stimulated an immediate interaction between PKC*α* and TNFR1 (Figure 2(e)), which was inhibited by pretreatment with Gö6976 (Figure 2(f)). These results suggested that TNF-*α*-induced cPLA_2_ expression is mediated through a TNFR1/PKC*α* signaling pathway in RASFs.

### 3.3. Involvement of NOX/ROS Generation in TNF-*α*-Induced cPLA_2_ Expression

**Figure 3:**
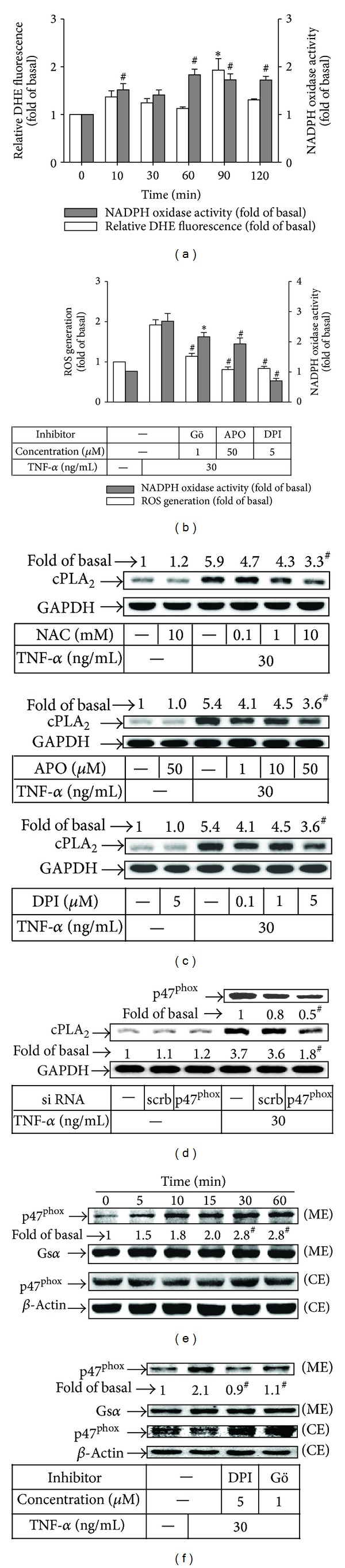
TNF-*α* induces NOX/ROS generation and cPLA_2_ expression via PKC*α*. (a) Cells were incubated with 30 ng/mL of TNF-*α* for the indicated time intervals. Next, the cells were added with (5 *μ*M) DHE and the number of DHE-labeled red-colored cells was counted under a fluorescence microscope. Open bars indicate statistical analysis of DHE staining. NADPH oxidase activity was determined (shaded bars). (b) RASFs were pretreated with Gö6976 (1 *μ*M), DPI (5 *μ*M), or APO (50 *μ*M) for 1 h and then stimulated with TNF-*α* for 90 min (open bars) or 1 h (shaded bars). ROS generation and NADPH oxidase activity were determined. (c) Cells were pretreated with NAC, DPI, or APO for 1 h and then incubated with TNF-*α* for 16 h. The expression of cPLA_2_ was determined by Western blotting. (d) Cells were transfected with scrambled or p47^phox^ siRNA and then incubated with TNF-*α* for 16 hours. The levels of p47^phox^ and cPLA_2_ protein were determined by Western blotting. Cells were incubated with (e) TNF-*α* for the indicated time intervals or (f) pretreated with DPI (5 *μ*M) or Gö6976 (Gö, 1 *μ*M) for 1 h and then incubated with TNF-*α* for 60 min. The membrane (ME) and cytosolic (CE) fractions were prepared and subjected to Western blot using an anti-p47^phox^ antibody. Values are the mean ± SEM of samples from 4 RA patients from 3 independent experiments. In (a) and (e), *P < 0.05; ^#^ *P* < 0.01 versus vehicle alone. In (b), (c), (d), and (f), *P < 0.05; ^#^ *P* < 0.01 versus TNF-*α* alone.

ROS are released during the inflammatory responses of joint tissues and associated with cartilage degradation in RA [6, 25, 26]. TNF-*α* induces expression of several genes mediated through NOX-dependent ROS intermediaries including H_2_O_2_ and superoxide anion [11]. First, we measured whether TNF-*α* could induce intracellular ROS production. As shown in Figure 3(a), TNF-*α* induced a significant increase in NOX activity and ROS production. PKC isoforms, mainly, PKC*α*, *β*II, and *δ*, have been characterized as an important activator of NOX [27, 28]. Here, we also showed that pretreatment with the inhibitors of PKC*α* (Gö6976) and NOX (DPI and APO) markedly reduced TNF-*α*-induced NOX activity and ROS levels (Figure 3(b)), suggesting that TNF-*α* induced ROS generation via PKC*α*/NOX in RASFs. We further established that TNF-*α* induced cPLA_2_ expression via NOX and ROS by using NAC, DPI, APO, or, p47^phox^ siRNA. As shown in Figures 3(c) and 3(d), pretreatment with NAC, DPI, or APO and transfection with p47^phox^ siRNA significantly abrogated TNF-*α*-induced cPLA_2_ expression. On the other hand, we observed that TNF-*α* time-dependently stimulated p47^phox^ translocation from the cytosol to the membrane, which was inhibited by pretreatment with DPI or Gö6976 (Figures 3(e) and 3(f)). Taken together, these data suggested that TNF-*α* induces cPLA_2_ expression via PKC*α*-dependent NOX activation and ROS generation in RASFs.

### 3.4. p38 MAPK- and JNK1/2-Dependent ROS Generation Is Involved in TNF-*α*-Mediated cPLA_2_ Expression

**Figure 4:**
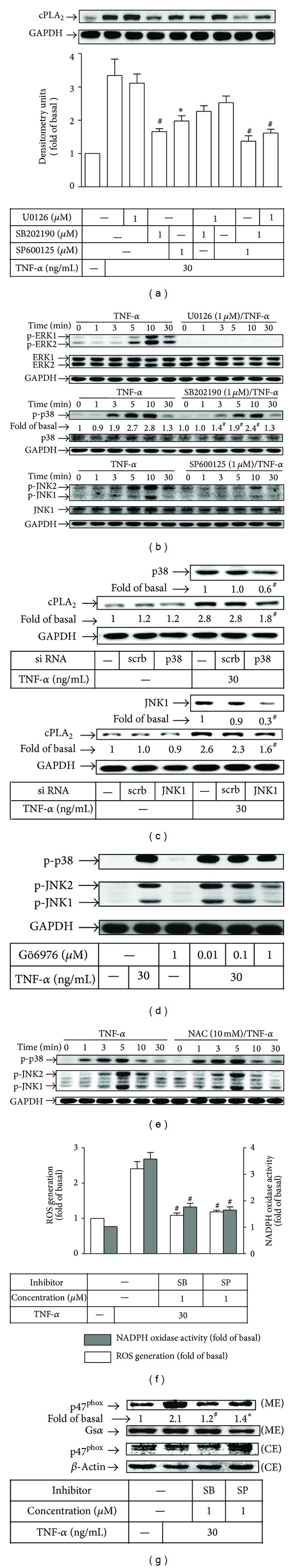
Involvement of PKC*α*-dependent p38 MAPK and JNK1/2 in TNF-*α*-mediated ROS generation and cPLA_2_ expression. (a) Cells were pretreated with U0126, SB202190, or SP600125, or combinatorial treatment for 1 h, and then incubated with TNF-*α* for 16 h. The expression of cPLA_2_ was determined by Western blotting. (b) Cells were pretreated with or without U0126, SB202190, or SP600125 for 1 h and then incubated with TNF-*α* for the indicated time intervals. (c) Cells were transfected with scrambled, p38, or JNK1 siRNA and then incubated with TNF-*α* for 16 h. The levels of p38, JNK1, and cPLA_2_ were determined by Western blotting. (d) Cells were pretreated with Gö6976 for 1 h and then stimulated with TNF-*α* for 30 min. The levels of phospho-p38 MAPK and phospho-JNK1/2 were determined by Western blotting. (e) Cells were pretreated with NAC for 1 h, and then incubated with TNF-*α* for the indicated time intervals. The levels of phospho-p38 MAPK and phospho-JNK1/2 were determined by Western blotting. (f) Cells were pretreated with SB202190 (1 *μ*M) or SP600125 (1 *μ*M) for 1 h before exposure to TNF-*α* for 1 h (shaded bars) or 2 h (open bars). The NOX activity (shaded bars) and ROS generation (open bars) were analyzed. (g) Cells were pretreated with SB202190 (1 *μ*M) or SP600125 (1 *μ*M) for 1 h before exposure to TNF-*α* for 1 h. The membrane (ME) and cytosolic (CE) fractions were prepared and subjected to Western blotting using an anti-p47^phox^ antibody. All analyses were performed on samples from 4 RA patients. Results are representative of 3 independent experiments. Values in (a), (c), (f), and (g) are the mean ± SEM. *P < 0.05; ^#^ *P* < 0.01 versus TNF-*α* alone.

To examine whether MAPKs are involved in TNF-*α*-induced ROS production and cPLA_2_ expression, the inhibitors of MEK1/2 (U0126), p38 MAPK (SB202190), and JNK1/2 (SP600125) were used. As shown in Figure 4(a), TNF-*α*-enhanced cPLA_2_ expression was inhibited by pretreatment with SB202190 and SP600125, but not U0126. Moreover, we found that combination of SB202190 and SP600125 caused a more effective decrease of TNF-*α*-induced cPLA_2_ expression. TNF-*α* also significantly stimulated p42/p44 MAPK, p38 MAPK, and JNK1/2 phosphorylation, which was inhibited by pretreatment with their respective inhibitors U0126, SB202190, or SP600125 during the period of observation (Figure 4(b)). To further ensure the roles of p38 MAPK and JNK1/2 in TNF-*α*-induced cPLA_2_ expression, as shown in Figure 4(c), transfection with siRNA of p38 MAPK or JNK1 downregulated the expression of their respective proteins and subsequently attenuated TNF-*α*-induced cPLA_2_ expression. Furthermore, pretreatment with Gö6976 reduced TNF-*α*-stimulated p38 MAPK or JNK1/2 phosphorylation (Figure 4(d)). However, pretreatment with NAC had no effect on p38 MAPK or JNK1/2 phosphorylation (Figure 4(e)). We further investigated the roles of p38 MAPK and JNK1/2 in TNF-*α*-stimulated NOX activity and ROS generation. As shown in Figure 4(f), pretreatment with SB202190 or SP600125 significantly abrogated TNF-*α*-stimulated NOX activity and ROS generation. In addition, TNF-*α*-stimulated translocation of p47^phox^ from the cytosol to membrane was also attenuated by SB202190 or SP600125 (Figure 4(g)). These results suggested that TNF-*α*-stimulated NOX activation and ROS generation is mediated through p38 MAPK- and/or JNK-1/2-stimulated membrane translocation of p47^phox^ in RASFs.

### 3.5. TNF-*α*-Induced cPLA_2_ Upregulation Is Mediated via a ROS-Dependent NF-*κ*B Signaling

**Figure 5:**
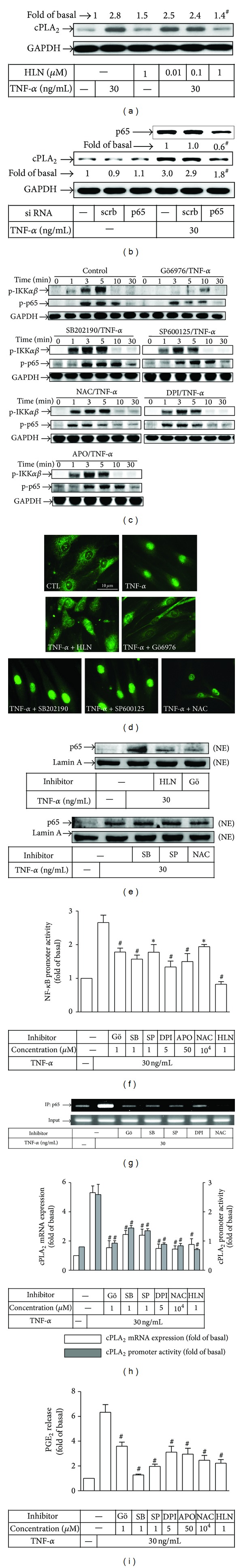
TNF-*α*-mediated ROS-dependent activation of NF-*κ*B is required for TNF-*α*-induced cPLA_2_ expression. (a) RASFs were pretreated with helenalin for 1 h and then incubated with TNF-*α* for 16 h. The expression of cPLA_2_ was determined by Western blotting. (b) Cells were transfected with scrambled or p65 siRNA and then incubated with TNF-*α* for 16 h. The levels of p65 and cPLA_2_ protein were determined by Western blotting. (c) Cells were pretreated with or without Gö6976 (1 *μ*M), SB202190 (1 *μ*M), SP600125 (1 *μ*M), or NAC (10 mM) for 1 h before exposure to TNF-*α* for the indicated time intervals. The levels of phospho-IKK*α*/*β* and phospho-p65 were determined by Western blotting. Cells were pretreated with Gö6976 (1 *μ*M), SB201290 (1 *μ*M), SP600125 (1 *μ*M), NAC (10 mM), or helenalin (1 *μ*M) for 1 h and then incubated with TNF-*α* for 30 min. (d) Cells were fixed and then labeled using an anti-p65 antibody and FITC-conjugated secondary antibody. (e) The nuclear extract (NE) was prepared and subjected to Western blotting using the indicated antibodies and lamin A used as an internal control. (f) Cells were transiently transfected with NF-*κ*B-Luc reporter gene, pretreated with Gö6976, SB201290, SP600125, DPI, APO, NAC, or helenalin for 1 h, and then incubated with TNF-*α* for 4 h. The promoter activity was determined. (g) Cells were pretreated with Gö6976, SB201290, SP600125, DPI, or NAC for 1 h and incubated with TNF-*α* for 2 h. Chromatin was immunoprecipitated using an anti-p65 antibody. The associated cPLA_2_ promoter DNA was amplified by polymerase chain reaction (PCR). Input represents PCR products from chromatin pellets before immunoprecipitation. (h) Cells were transfected without or with a cPLA_2_-Luc reporter gene, pretreated with Gö6976, SB201290, SP600125, DPI, APO, NAC, or helenalin for 1 h, and then incubated with TNF-*α* for 6 h. The mRNA levels of cPLA_2_ (open bars) and cPLA_2_-luc promoter (shaded bars) were measured. (i) The PGE_2_ levels in the media were analyzed using a PGE_2_ EIA kit. All analyses were performed on samples from 4 RA patients. Results are representative of 3 independent experiments. Values in (a), (b), (e), (f), and (g) are the mean ± SEM. *P < 0.05; ^#^ *P* < 0.01 versus TNF-*α* alone.

NF-*κ*B is activated by oxidative stress or cytokines and is critical to the expression of inflammatory genes [11, 29, 30]. We found that TNF-*α*-enhanced cPLA_2_ protein expression was inhibited by pretreatment with helenalin (an inhibitor of NF-*κ*B) or transfection with p65 siRNA (Figures 5(a) and 5(b)). Since NF-*κ*B signaling depends on activation of the IKK complex [31], we examined whether the PKC*α*-, JNK1/2-, p38 MAPK-, and ROS-dependent pathways are involved in TNF-*α*-mediated IKK*α*/*β* phosphorylation. As shown in Figure 5(c), TNF-*α* stimulated IKK*α*/*β* and p65 phosphorylation which were inhibited by Gö6976, but not by SB202190, SP600125, DPI, APO, or NAC. These responses were further confirmed by using immunofluorescence staining showing that pretreatment with helenalin or Gö6976 inhibited TNF-*α*-induced NF-*κ*B translocation, whereas SB202190, SP600125, or NAC had no effect on nuclear translocation of NF-*κ*B (Figure 5(d)). Similar results were obtained with isolated nuclear fraction from RASFs determined by Western blot (Figure 5(e)), suggesting that activation of the IKK complex results in the phosphorylation and the nuclear translocation of p65, which is independent of p38 MAPK, JNK1/2, and ROS. We further found that pretreatment with Gö6976, SB202190, SP600125, DPI, APO, NAC, or helenalin suppressed TNF-*α*-induced NF-*κ*B promoter activity (Figure 5(f)). Moreover, the recruitment of nuclear NF-*κ*B p65 DNA-binding activity to cPLA_2_ promoter detected by chromatin immunoprecipitation assay (ChIP) was consistent with NF-*κ*B luciferase reporter activity (Figure 5(g)), indicating that NF-*κ*B transcriptional activity is involved in cPLA_2_ expression mediated through activation of PKC*α*, p38 MAPK, and JNK1/2 following the generation of ROS in RASFs. Finally, we showed that pretreatment with Gö6976, SB202190, SP600125, DPI, NAC, or helenalin attenuated TNF-*α*-induced cPLA_2*α*_ mRNA expression and luciferase promoter activity (Figure 5(h)). In addition, pretreatment with these inhibitors also attenuated TNF-*α*-induced PGE_2_ synthesis (Figure 5(i)), suggesting that the PKC*α*-dependent activation of p38 MAPK, JNK1/2, NOX/ROS generation, and NF-*κ*B participates in TNF-*α*-induced cPLA_2_ expression and PGE_2_ production in RASFs.

### 3.6. Regulation of cPLA_2_ Expression in TNF-*α*-Treated Mice

**Figure 6:**
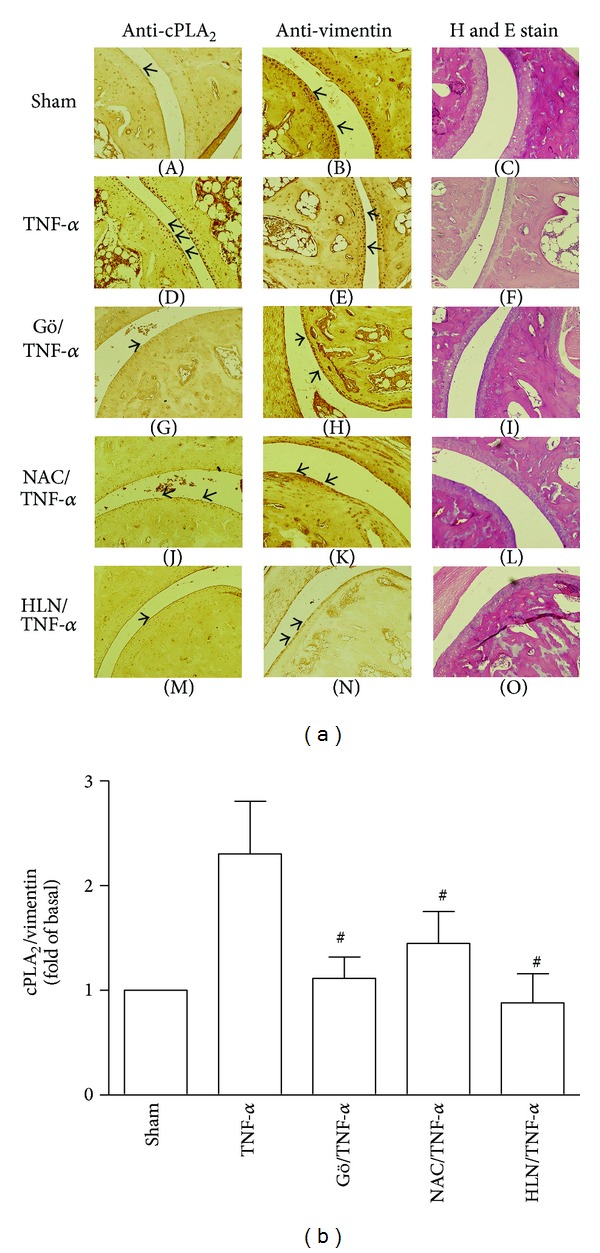
Regulation of cPLA_2_ expression in TNF-*α*-treated mice. (a) Immunohistochemical staining for cPLA_2_ and vimentin and hematoxylin and eosin (H&E) staining in serial sections of ankle joints from PBS-treated mice (sham; (A)–(C)), TNF-*α*-injected mice (TNF-*α*; (D)–(F)), Gö6976-pretreated mice (Gö/TNF-*α*; (G)–(I)), NAC-pretreated mice (NAC/TNF-*α*; (J)–(L)), and helenalin-pretreated mice (HLN/TNF-*α*; (M)–(O)) are shown. Microscopic observation showed vimentin in the synovial membrane of ankle joints, and synovial fibroblasts overlapping with cPLA_2_ expression. Arrowheads indicate positive staining. (b) The diagrammatic representation of quantitative data of cPLA_2_-positive cells in the articular joints of mice injected with the indicated inhibitors. Results are representative of 3 mice per experimental group. In (b), values are the mean ± SEM. ^#^ *P* < 0.01, as compared with the cells exposed to TNF-*α* alone.

To further confirm our *in vitro* results, we tested the effect of CORM-2 on the expression of cPLA_2_ and HO-1 in the ankle joints of mice challenged with TNF-*α* *in vivo*. As shown in Figure 6(a)-(D), the synovial layer in TNF-*α*-treated ankle joints strongly expressed cPLA_2_, which was reduced by pretreatment with Gö6976, NAC, or helenalin (Figure 6(a)-(G), J, and M). The quantitative data of immunohistochemical staining (Figure 6(b)) demonstrated that TNF-*α*-induced cPLA_2_ expression occurs both *in vitro* and *in vivo*, which is mediated via PKC*α*-dependent NOX activation/ROS generation and NF-*κ*B activation.

### 3.7. Suppressive Effects of CO-RM2 on ROS Generation and Activity of NF-*κ*B Induced by TNF-*α* in RASFs

**Figure 7:**
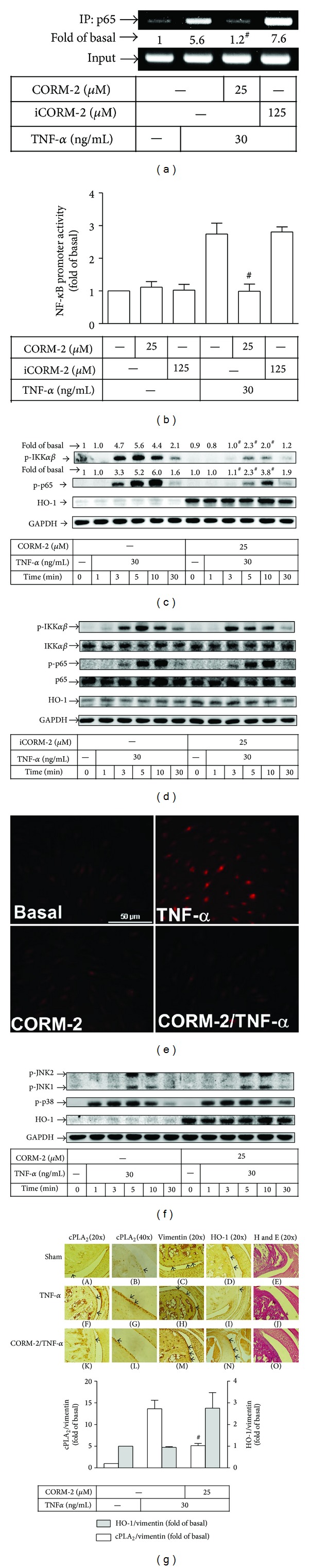
Effect of CORM-2 on TNF-*α*-induced NF-*κ*B activation and cPLA_2_ expression. (a) Cells were pretreated with CORM-2 or iCORM-2 for 16 h and then incubated with TNF-*α* for 2 h. Chromatin immunoprecipitation (ChIP) assays were performed using an anti-p65 antibody. (b) Cells were transfected with NF-*κ*B-luc reporter gene, pretreated with or without CORM-2 for 16 h, and then incubated with TNF-*α* for 4 h. NF-*κ*B promoter activity was determined. ((c), (d), and (f)) Cells were pretreated with or without CORM-2 or iCORM-2 for 16 h and then treated with TNF-*α* for the indicated time intervals. The levels of phospho-p65, phospho-IKK*α*/*β*, phospho-JNK1/2, phospho-p38 MAPK, and HO-1 were determined by Western blotting. (e) Cells were pretreated with or without CORM-2 for 16 h and then incubated with TNF-*α* for 90 min. The cells were added with (5 *μ*M) DHE and images acquired to determine the ROS generation using a fluorescence microscopy. All analyses were performed on samples from 4 RA patients. (f) Immunohistochemical staining for HO-1, cPLA_2_, or vimentin and hematoxylin and eosin (H&E) staining of serial sections of ankle joints from mice injected with phosphate-buffered saline (sham) ((A)–(E)), mice injected with TNF-*α* ((F)–(J)), and mice injected with CORM-2 for 16 h followed by TNF-*α* for 24 h ((K)–(O)) were performed. Arrowheads indicate positive staining. Results are representative of 3 mice per experimental group. Results are representative of 3 independent experiments. In (a), (b), (c), and (g), values are the mean ± SEM. *P < 0.05; ^#^ *P* < 0.01 versus TNF-*α* alone.

We have shown that CO-RM2 inhibited TNF-*α*-induced cPLA_2_ expression via HO-1 induction in RASFs (Figure 1). As upregulation of cPLA_2_ expression depends on activation of NF-*κ*B, we next assessed whether CO-RM2 interfered with these processes. Chromatin was immunoprecipitated using an anti-p65 antibody, and the cPLA_2_ promoter region was amplified by PCR. As shown in Figure 7(a), TNF-*α*-induced p65 binding to the cPLA_2_ promoter was inhibited by pretreatment with CO-RM2, but not iCO-RM2. Moreover, exposure to TNF-*α* increased NF-*κ*B promoter activity which was attenuated by pretreatment with CO-RM2 (Figure 7(b)). The classical NF-*κ*B is activated by I*κ*B*α* degradation, which occurs subsequent to IKK*α*/*β* phosphorylation. To investigate whether the inhibition of NF-*κ*B promoter activity was due to the inhibition of IKK*α*/*β* and p65 phosphorylation, as shown in Figures 7(c) and 7(d), TNF-*α*-stimulated IKK*α*/*β* and p65 phosphorylation was attenuated by CO-RM2 but not iCO-RM2, during the period of observation. In addition, we demonstrated that TNF-*α*-stimulated generation of ROS was responsible for NF-*κ*B transcriptional activity which was inhibited by pretreatment with CORM-2 (Figure 7(e)). On the other hand, CORM-2 had no effect on TNF-*α*-induced phosphorylation of p38 MAPK and JNK1/2 in RASFs (Figure 7(f)). These data demonstrated that HO-1 induction by CO-RM2 attenuates TNF-*α*-induced cPLA_2_ expression mediated through suppression of ROS and NF-*κ*B. To confirm these results in *in vivo* studies, mice were intra-articularly administered with CO-RM2 for 16 h and then followed with TNF-*α* for 24 h. The images of immunohistochemical staining in the articular joints showed that the number of cPLA_2_-expressing cells was significantly higher in TNF-*α*-treated mice than those of PBS-treated mice (Figure 7(g)-(A, F)). Administration with CO-RM2 before TNF-*α* treatment resulted in decreased cPLA_2_ expression on synovial layer in the articular joints of mice (Figure 7(g)-(F, K)). The levels of cPLA_2_ expression normalized to vimentin are summarized in the bar graph (Figure 7(g), lower panel). These results suggested that CO-RM2 attenuates TNF-*α*-induced cPLA_2_ expression in the articular joints of mice.

## 4. Discussion

**Figure 8:**
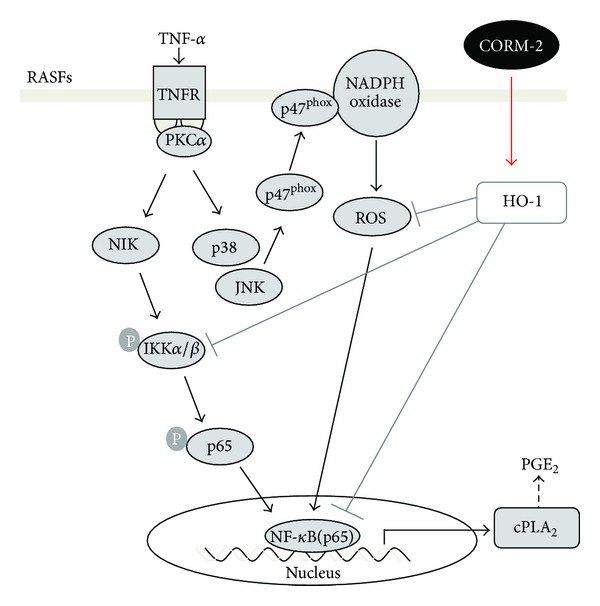
Effects of CORM-2 on the TNF-*α*-induced cPLA_2_ expression. TNF-*α* stimulated NF-*κ*B activation through TNFR1/PKC*α*/IKK*α*/*β*, and p38 MAPK-, JNK-1/2-dependent NOX/ROS pathways resulting in cPLA_2_ expression in RASFs. The downregulation of TNF-*α*-induced cPLA_2_ expression by CORM-2 is mediated by the reduction in NF-*κ*B transcriptional activity and decrease in generation of NOX/ROS.

Inflammation and oxidative stress play a key role in the pathogenesis of RA. cPLA_2_ may represent a pathogenic link between the generation of eicosanoids and the production of inflammatory molecules in the development of arthritis [5]. CO-RM has been shown to perform anti-inflammatory effects in various cell types [11, 16, 32]. Thus, in this study, we attempted to investigate the protective mechanisms of CORM-2 in TNF-*α*-challenged RASFs and ICR mice. Here, we demonstrated that TNF-*α*-induced cPLA_2_ expression was regulated via a complex of TNFR1/PKC*α* that triggered the activation of p38 MAPK- and JNK1/2-dependent NOX/ROS generation, leading to activation of NF-*κ*B in RASFs. Moreover, we found that CORM-2 hampered p65 recruitment to the promoter of cPLA_2_ through the attenuation of IKK*α*/*β* and p65 phosphorylation and ROS production, leading to the suppression of TNF-*α*-induced cPLA_2_ expression (Figure 8).

Synovial fibroblasts have been shown to express the classical PKC*α*, which is DAG and Ca^2+^ dependent, and PKC*δ*, which does not require either DAG or Ca^2+^ [33]. PKC*δ* associates with the TNFR1 complex after TNF stimulation [34], and PKCs are enriched in lipid rafts where the engaged TNFR1 complex resides [35]. In RASFs, we established that TNF-*α* induced cPLA_2_ expression via a PKC*α*/*β* signaling. TNF-*α* could directly stimulate PKC*α* and PKC*β*II translocation. We further investigated the physical association of TNFR1 and PKC*α* in TNF-*α*-induced cPLA_2_ expression. Although the detail protein-protein interactions among TNFR1 and PKC*α* are not known, our results are the first time to show a role of TNFR1/PKC*α* complex formation in TNF-*α*-induced cPLA_2_ expression in RASFs.

TNF-*α* induces expression of several genes indirect through short-lived ROS intermediaries including H_2_O_2_ and superoxide anion [11, 36]. The biological function of NOX enzymes might contribute to the production of ROS [37]. Activated NOX is a multimeric protein complex consisting of at least three cytosolic subunits of p47^phox^, p67^phox^, and p40^phox^. The p47^phox^ regulatory subunit plays a critical role in acute activation of NOX; phosphorylation of p47^phox^ is thought to relieve the inhibitory intracellular interactions and permit the binding of p47^phox^ to p22^phox^, thereby increasing oxidase activation [11, 37]. Here, we established that TNF-*α* induced cPLA_2_ expression via a p47^phox^/NOX-dependent ROS pathway in RASFs.

PKC isoforms, mainly PKC*α* and *β*II, have been characterized as an important activator of NOX [27, 38]. It has also been found that PKC*α*, but not PKC*β*, is required for NOX activation [39]. However, we observed that inhibition of PKC*α*/*β* markedly reduced TNF-*α*-mediated NOX activation and ROS generation in RASFs. Thus, we suggested that, in RASFs, PKC*α*/*β* play key roles in mediating ROS-dependent cPLA_2_ expression. In addition, a previous study suggested an important role of ROS in TNF-induced MAPKs activation [40]. It has been demonstrated that ROS can induce or mediate the activation of these MAPKs pathways, indicating the involvement of ROS in activation of MAPKs [11, 41]. However, under our experimental conditions, we found that JNK1/2 and p38 MAPK were involved in TNF-*α*-induced NOX activation and ROS generation in RASFs. Similar to study of Pandy and Fulton, they have shown that MAPKs can phosphorylate the regulatory subunits of NOX enzymes and induce ROS generation [42]. The regulatory subunit p47^phox^, which can modulate the activities of NOX1, 2, and 3, has been shown to be phosphorylated by Erk1/2 leading to an increase in NOX2 activity [42]. It has also been reported that TNF-*α*-induced ROS accumulation mediates prolonged MAPKs activation and cell death in mouse embryonic fibroblasts [43]. Moreover, it is believed that the ROS-thioredoxin-ASK1 system serves as the molecular switch that converts redox signal to JNK kinase activation [44]. This conclusion is based on observations that TNF-*α*-induced ROS generation was found only in wild-type (WT) mouse fibroblasts but not in JNK^−/−^ cells [45]. Although the conventional dogma places ROS upstream of MAPKs activation, it is noteworthy that a recent study points out a positive feedback loop between MAPKs activation and ROS production. In the present study, we also confirmed that TNF-*α*-stimulated p38 MAPK and JNK1/2 phosphorylation was not attenuated by NAC in RASFs. Thus, we suggested that p38 MAPK and JNK1/2 are the upstream mediators which can regulate p47^phox^ translocation and ROS generation in RASFs.

NF-*κ*B exerts its functions by regulating the transcription of genes encoding many immunoregulators, inflammatory mediators, and inhibitors of apoptosis. Several studies have also underscored the key role of the IKK/NF-*κ*B pathway in the induction and maintenance of the state of inflammation [46]. PKC*α* has been shown to be involved in TNFR-mediated NF-*κ*B signaling [24]. In the current study, we found that TNF-*α* induced IKK*α*/*β* and NF-*κ*B activation via a PKC*α*, but not p38 MAPK, JNK1/2, and ROS in RASFs. Since activation of MAPKs was involved in TNF-*α*-stimulated cPLA_2_ expression at transcriptional level [14], we also examined whether the NF-*κ*B-dependent transcriptional activity was regulated by the phosphorylation of MAPKs. Here, we found that inhibition of p38 MAPK, JNK1/2, and NOX/ROS attenuated NF-*κ*B promoter activity and recruitment of p65 interacted with cPLA_2_ promoter, implying that the activation of p38 MAPK- and JNK1/2-dependent NOX/ROS generation was required for the TNF-*α*-induced NF-*κ*B transcriptional activity.

HO-1 is an enzyme that catalyzes the degradation of heme, which produces biliverdin, iron, and CO. These by-products have been implicated in the cytoprotective responses against oxidative stress [11, 15, 16]. In addition to the antioxidant activities of biliverdin, it has been shown that CO inhibits the expression of LPS-induced proinflammatory cytokines in macrophages, suggesting that CO is involved in the anti-inflammatory effect action of HO-1 [47]. Recent studies have demonstrated that CO is the key molecule mediating the protective effect of HO-1. Therefore, CO-RMs are now being used as useful pharmacological tools for investigation of CO effect [48]. However, the effects and potential mechanisms of CORM-2 in modulation of TNF-*α*-induced cPLA_2_ expression in RASFs remain to be clarified.

CORM-2 attenuates expression of ICAM-1 protein by interfering with NF-*κ*B activation in renal tissues [33]. It also inhibited chemokine production induced by IL-1*β* in OA synoviocytes [16]. Our data clearly show that CORM-2 is a potent inducer of HO-1 and exhibits inhibitory effects on TNF-*α*-induced cPLA_2_ expression in RASFs. The downregulation of cPLA_2_ mRNA/protein levels by CORM-2 could be mediated by the reduction of NF-*κ*B transcriptional activity. Previous studies have shown that CORM-2 decreases ROS production and NF-*κ*B activation induced by cytokines in OA synoviocytes [16]. Indeed, we found that CORM-2 also inhibited TNF-*α*-regulated p65 and IKK*α*/*β* phosphorylation and ROS generation. Thus, we suggested that CORM-2 has a protective effect against TNF-*α*-triggered inflammatory responses. It has been reported that CORM-2 played a regulatory role in phosphorylation of Erk1/2 and JNK1/2 in OA synoviocytes [16]. However, in RASFs, CORM-2 failed to attenuate p38 MAPK and JNK1/2 phosphorylation. Therefore, the anti-inflammatory effects of CORM-2 on RASFs occur, at least in part, via its ability to attenuate oxidative stress and NF-*κ*B transcriptional activity which could participate in its inhibitory effects on cPLA_2_ expression induced by TNF-*α*. In addition, we confirmed that CORM-2 mediates the inhibitory effects of HO-1 on TNF-*α*-induced cPLA_2_ expression via CO. Our data show that iCORM-2, which does not liberate CO, fails to induce HO-1 expression and to reduce TNF-*α*-induced cPLA_2_ expression in RASFs. In contrast, TNF-*α*-induced cPLA_2_ expression was slightly enhanced upon HO-1 knockdown.

We found that CORM-2 attenuated cPLA_2_ but not COX-2 (data not shown) expression mediated through suppression of NF-*κ*B activation. It is consistent with others that CORM-2 reduced iNOS expression but not COX-2 in LPS-induced RAW 264.7 cells [48]. Conversely, Guillén et al. [32] have documented that CORM-2 is able to downregulate COX-2 expression and PGE_2_ production through the inhibition of NF-*κ*B activation in IL-1*β*-stimulated osteoarthritic chondrocytes, while the modulation of COX-2 mRNA expression was not significant. Similarly, we found that CORM-2 alone increased COX-2 mRNA expression within 16 h (data not shown). In this study, CORM-2 significantly attenuated TNF-*α*-induced cPLA_2_ expression but not COX-2 expression and PGE_2_ production. The effect of CORM-2 on cytokines-induced COX-2 expression and PGE_2_ production is still controversial and these effects on various cell types may be due to different experimental conditions and cell types.

## 5. Conclusions

In summary, as depicted in Figure 8, TNF-*α* induced NF-*κ*B activation through TNFR1/PKC*α*/IKK*α*/*β* and p38 MAPK- and JNK-1/2-dependent NOX/ROS pathways leading to cPLA_2_ expression in RASFs. We revealed the TNFR1/PKC*α*-dependent participation of IKK*α*/*β* and NOX/ROS pathways on translocation and DNA-binding ability of NF-*κ*B in TNF-*α*-challenged RASFs. Moreover, we have demonstrated for the first time that the downregulation of cPLA_2_ protein/mRNA by CORM-2 could be mediated by the reduction in NF-*κ*B transcriptional activity which would be dependent on the inhibition of IKK*α*/*β* phosphorylation leading to attenuation of nuclear translocation of NF-*κ*B. These results elucidate the molecular mechanisms underlying the pharmacological effects of CORM-2 and may lead to the development of novel therapeutic strategies of RA.

## Glossary

TNF-*α*: Tumor necrosis factor-*α*
cPLA_2_: Cytosolic phospholipase A_2_
RASFs: Rheumatoid arthritis synovial fibroblasts
TNFR1: TNF receptor l
MAPKs: Mitogen-activated protein kinases
ERK: Extracellular-regulated protein kinase
JNK: c-Jun-N-terminal kinase
siRNA: Small interfering RNA
RT-PCR: Reverse transcription-polymerase chain reaction
NF-*κ*B: Nuclear factor-kappa B.
ROS: Reactive oxygen species
NOX: NADPH oxidase
HO-1: Heme oxygenase 1
CORM-2: Carbon monoxide releasing molecule-2.
